# Diagnostic Accuracy of p16 Immunohistochemistry as a Marker of High-Risk HPV in Invasive Laryngeal Squamous Cell Carcinoma: A Systematic Review

**DOI:** 10.3390/medicina62071372

**Published:** 2026-07-16

**Authors:** Ana-Maria Stanoiu, Delia Hutanu, Maria Sorop-Florea, Alexandru Alexandru, Norberth-Istvan Varga, Iulia Cristina Bagiu, Mihaela-Diana Popa, Bogdan Hirtie, Nicolae-Constantin Balica, Cristian-Ion Mot, Ioana-Delia Horhat

**Affiliations:** 1 Doctoral School, “Victor Babes” University of Medicine and Pharmacy, Eftimie Murgu Square, No. 2, 300041 Timisoara, Romania; 2 Department Biology-Chemistry, Faculty of Chemistry-Biology-Geography, West University of Timisoara, Pestalozzi, 16, 300315 Timisoara, Romania; 3 Faculty of Medical and Behavioral Sciences, “Constantin Brancusi” University, Tineretului Street, No. 4, 210185 Targu Jiu, Romania; 4 Department of Nursing, “Victor Babes” University of Medicine and Pharmacy, Eftimie Murgu Square, No. 2, 300041 Timisoara, Romania; 5 Department of Microbiology, “Victor Babes” University of Medicine and Pharmacy, Eftimie Murgu Square, No. 2, 300041 Timisoara, Romania; 6 Department of Ear Nose Throat, “Victor Babes” University of Medicine and Pharmacy, Eftimie Murgu Square, No. 2, 300041 Timisoara, Romania

**Keywords:** p16 immunohistochemistry, human papillomavirus, high-risk HPV, laryngeal squamous cell carcinoma, laryngeal cancer, diagnostic accuracy, surrogate marker, HPV-driven carcinogenesis, RNA in situ hybridization

## Abstract

*Background and Objectives*: p16 immunohistochemistry (IHC) is widely used as a surrogate marker for high-risk human papillomavirus (HPV)-driven carcinogenesis in oropharyngeal squamous cell carcinoma. Diagnostic reliability in laryngeal squamous cell carcinoma (LSCC) is still up for debate, particularly because HPV DNA detection, p16 overexpression, and transcriptionally active HPV infection may be discordant at this anatomical site. This systematic review aimed to assess the diagnostic performance of p16 IHC as a surrogate marker for high-risk HPV status in primary invasive LSCC. *Materials and Methods*: A systematic review was conducted in accordance with PRISMA principles and prospectively registered in PROSPERO. PubMed, Scopus, and Web of Science, and citation searches were used to identify studies reporting paired p16 IHC and tumour-based molecular HPV testing in invasive LSCC. Eligible HPV reference standards included HPV DNA PCR/genotyping, DNA in situ hybridization, RNA in situ hybridization, and E6/E7 mRNA detection. Data were synthesized narratively because of substantial heterogeneity in p16 thresholds, HPV assays, and study populations. *Results*: Fourteen studies were included. p16 positivity thresholds varied widely, ranging from ≥30% moderate/strong staining to ≥70–75% diffuse nuclear and cytoplasmic staining. HPV reference standards also differed substantially across studies. Overall, p16 IHC showed inconsistent concordance with molecular HPV testing. Studies using RNA-based reference standards showed that transcriptionally active HPV was uncommon in LSCC and that p16-positive tumours often lacked evidence of active HPV transcription. *Conclusions*: p16 IHC should not be used as a standalone surrogate marker for high-risk HPV-driven carcinogenesis in invasive LSCC. When HPV attribution is clinically or analytically important, p16-positive cases should be confirmed using HPV-specific molecular testing, preferably RNA-based assays. Future prospective studies using standardized p16 protocols and transcriptionally active HPV reference standards are needed.

## 1. Introduction

Squamous cell carcinoma (SCC) of the larynx represents the most common malignancy of the laryngopharyngeal region, with approximately 184,000 new cases and 100,000 deaths reported annually worldwide, predominantly affecting men with a history of tobacco and alcohol exposure [1,2]. While the pathogenesis of laryngeal SCC is driven predominantly by carcinogen-related mutagenesis, a proportion of cases has been associated with high-risk human papillomavirus (HPV) infection—a finding with potential implications for tumour biology, prognosis, and treatment. Central to the clinical management of this subset is the use of p16 immunohistochemistry (IHC) as a surrogate marker for HPV-driven carcinogenesis, a practice increasingly applied in head and neck oncology despite uncertainty about its validity at this anatomical site [3,4].

p16 (encoded by *CDKN2A*, also designated p16^INK4a^) is a cyclin-dependent kinase inhibitor that restrains cell cycle progression through inhibition of CDK4/6-mediated phosphorylation of the retinoblastoma protein (pRb) [5,6]. It is important to distinguish three non-equivalent readouts that are often conflated: HPV DNA detection (which indicates viral presence but not necessarily transforming activity), p16 overexpression (a downstream host response that can also arise HPV-independently), and detection of E6/E7 mRNA or RNA in situ hybridization (which reflects transcriptionally active, biologically relevant infection). In the setting of high-risk HPV infection, the viral oncoprotein E7 binds and functionally inactivates pRb, releasing E2F transcription factors and secondarily inducing compensatory upregulation of p16 through disruption of the pRb–E2F regulatory axis [7,8,9,10]. This mechanism provides the biological rationale for interpreting diffuse, strong p16 overexpression as a surrogate readout of transcriptionally active HPV infection [11]. In oropharyngeal SCC, where HPV—predominantly HPV16—is the dominant oncogenic driver in high-income countries, this surrogate relationship is well-validated and has been formally incorporated into the American Joint Committee on Cancer (AJCC) 8th Edition staging system as the defining criterion for HPV-associated oropharyngeal SCC [12,13,14,15]. Pooled estimates from systematic reviews report sensitivity exceeding 94% and specificity of approximately 90% for p16 IHC relative to tumour-based HPV reference standards in this setting [11,14,16].

Meta-analytic data indicate that HPV DNA has been detected in a variable but substantial proportion of laryngeal tumours—with pooled estimates in the range of 20–25% in some series—though HPV DNA has also been found in histologically normal laryngeal mucosa, raising important questions regarding the biological relevance of HPV detection at this anatomical site [4,17]. Studies applying transcription-based reference standards—including E6/E7 mRNA detection and RNA in situ hybridization—consistently find that the proportion of laryngeal SCCs attributable to biologically active HPV infection is substantially lower than estimates derived from HPV DNA prevalence or p16 positivity alone, suggesting that HPV DNA detection in laryngeal tissue frequently represents non-transforming or incidental infection [18,19,20]. Across multiple cohorts, transcriptionally active HPV has been identified in only a small minority of tumours, while discordance between HPV DNA, p16 expression, and viral transcriptional activity has been repeatedly observed [20,21]. Several studies have further demonstrated poor concordance between p16 immunohistochemistry and HPV status in LSCC, highlighting the limited reliability of p16 as a surrogate marker outside the oropharynx [22,23,24]. Collectively, current evidence suggests that HPV contributes causally to only a small subset of laryngeal SCCs, likely fewer than 5%, supporting the view that HPV-associated laryngeal carcinogenesis is biologically distinct from the established paradigm in oropharyngeal SCC [18,25].

This pattern of discordance between p16 overexpression and true HPV activity in laryngeal SCC is biologically plausible. Unlike the oropharynx, where lymphoid-type epithelium harbours a specific susceptibility to HPV-driven transformation, the stratified squamous epithelium of the larynx is predominantly transformed by tobacco- and alcohol-related mutagenesis [26,27]. In this context, p16 overexpression may arise through HPV-independent mechanisms—including alterations in the CDK–RB–E2F regulatory axis unrelated to viral oncogenesis—producing a pattern of false-positive p16 staining relative to true HPV activity that would not be observed in HPV-driven tumour sites [28,29].

The clinical implications of this diagnostic uncertainty are increasingly significant. As treatment strategies for HPV-associated head and neck tumours continue to be refined, and as molecular tumour subtyping informs multidisciplinary decision-making, the accurate identification of truly HPV-driven laryngeal tumours becomes essential [30]. Misclassifying a tobacco-driven laryngeal SCC as HPV-positive based on p16 alone could expose patients to inappropriate management pathways, while failing to identify a truly HPV-driven tumour may deny patients relevant prognostic information and eligibility for emerging de-escalation strategies [11,31].

Individual studies report highly variable sensitivity and specificity estimates, with heterogeneity in p16 positivity thresholds, HPV reference standard types, and population characteristics precluding straightforward cross-study comparison [11,30,32].

This systematic review was conducted to appraise the evidence on the diagnostic accuracy of p16 IHC relative to tumour-based molecular HPV reference standards in invasive laryngeal SCC, to characterize patterns of p16/HPV concordance and discordance, and to identify sources of methodological heterogeneity that may explain variability in reported performance estimates.

## 2. Materials and Methods

### 2.1. Protocol and Registration

This systematic review was conducted and reported in accordance with the Preferred Reporting Items for Systematic Reviews and Meta-Analyses extension for Diagnostic Test Accuracy [33]. The review protocol was prospectively registered in the International Prospective Register of Systematic Reviews (PROSPERO; CRD420261294225) prior to data extraction. No ethical approval was required, as the review is based exclusively on previously published data.

### 2.2. Eligibility Criteria (PICOS)

Eligible studies enrolled human patients with histologically confirmed primary invasive squamous cell carcinoma (SCC) of the larynx (including glottic, supraglottic, and subglottic subsites), of any clinical stage, treated at any institution. Studies were excluded if they enrolled only pre-invasive lesions (e.g., laryngeal dysplasia or carcinoma in situ without an invasive component), or if the laryngeal cases could not be separated from a mixed head-and-neck cohort in which extractable larynx-specific data were unavailable.

The index test was p16 immunohistochemistry (IHC) performed on primary laryngeal tumour tissue (biopsy or surgical resection specimen) using any antibody clone or automated platform, provided that a clearly stated positivity criterion was reported (e.g., block-type/diffuse staining, percentage threshold, or combined intensity-distribution scoring). Studies applying p16 IHC to cytological specimens only were excluded.

The reference standard was a tumour-based high-risk HPV molecular assay applied to the same specimen or a paired sample from the same primary tumour. Eligible assay types included the following: high-risk HPV DNA detection by polymerase chain reaction (PCR) or PCR-based genotyping; high-risk HPV DNA in situ hybridization (DNA ISH); high-risk HPV RNA in situ hybridization (RNA ISH), including RNAscope; and HPV E6/E7 mRNA detection by reverse-transcription PCR (RT-PCR). Studies in which HPV status was defined exclusively by p16 IHC—without an independent molecular assay—were excluded to avoid incorporation bias. When multiple HPV assays were applied to the same cases, the following pre-specified hierarchy was used to select the primary dataset: (1) E6/E7 mRNA assay (RT-PCR or RNA ISH); (2) HPV DNA ISH; (3) HPV DNA PCR/genotyping. Data from non-primary assays were retained for sensitivity analyses.

The primary outcomes were the diagnostic accuracy parameters of p16 IHC for the detection of high-risk HPV positivity in laryngeal SCC. Where formal pooling was not feasible due to heterogeneity, concordance between p16 and HPV status was synthesized narratively, stratified by reference standard type.

Cross-sectional, prospective cohort, retrospective cohort, and case–control studies were eligible, provided they contained paired p16 IHC and molecular HPV data on the same tumour specimens. Case reports and case series with fewer than ten laryngeal SCC specimens, narrative reviews, editorials, conference abstracts without sufficient extractable data, animal studies, and laboratory studies were excluded.

Studies in languages other than English were excluded.

### 2.3. Search Strategy

Comprehensive systematic literature searches were executed across three primary electronic databases from their inception and up to March 2026: PubMed, Scopus, and Web of Science. Supplementary searches were performed using Google Scholar to identify grey literature and records potentially missed by controlled-vocabulary indexing, following established guidelines for exhaustive systematic searching [34]. To ensure citation closure, manual backward citation screening was performed on all eligible articles, alongside forward citation chasing of index papers via Web of Science [35].

Structured Boolean search queries were developed for the laryngeal SCC population, combining terms for the anatomical site, p16/CDKN2A, IHC methodology, HPV, and diagnostic accuracy concepts. Queries were adapted for the syntax and controlled vocabulary of each database (MeSH for PubMed; topic field tags for Web of Science and Scopus).

The primary Scopus search strategy was as follows:

TITLE-ABS-KEY((“p16” OR “CDKN2A” OR “cyclin dependent kinase inhibitor 2A” OR “INK4a”) AND (immunohistochemistry OR immunostain* OR IHC) AND (“human papillomavirus” OR HPV) AND ((larynx OR laryngeal OR glottic OR supraglottic OR subglottic OR “laryngeal neoplasms”) AND (squamous OR “squamous cell carcinoma” OR SCC OR carcinoma))).

The primary Web of Science search strategy is the following:

TS = ((“p16” OR “CDKN2A” OR “cyclin dependent kinase inhibitor 2A” OR “INK4a”) AND (immunohistochemistry OR immunostain* OR IHC) AND (“human papillomavirus” OR HPV) AND ((larynx OR laryngeal OR glottic OR supraglottic OR subglottic OR “laryngeal neoplasms”) AND (squamous OR “squamous cell carcinoma” OR SCC OR carcinoma))).

The primary PubMed search strategy was as follows:

(“p16”[tiab] OR “CDKN2A”[tiab] OR “cyclin dependent kinase inhibitor 2A”[tiab] OR “INK4a”[tiab] OR “Genes, p16”[Mesh] OR “Cyclin-Dependent Kinase Inhibitor p16”[Mesh]) AND (immunohistochemistry[tiab] OR immunostain*[tiab] OR IHC[tiab] OR “Immunohistochemistry”[Mesh]) AND (“human papillomavirus”[tiab] OR HPV[tiab] OR “Papillomaviridae”[Mesh] OR “Alphapapillomavirus”[Mesh]) AND ((larynx[tiab] OR laryngeal[tiab] OR glottic[tiab] OR supraglottic[tiab] OR subglottic[tiab] OR “Laryngeal Neoplasms”[Mesh]) AND (squamous[tiab] OR “squamous cell carcinoma”[tiab] OR SCC[tiab] OR carcinoma[tiab] OR “Carcinoma, Squamous Cell”[Mesh])).

Deduplication was performed in Zotero reference-management software (version 9.0.5; Corporation for Digital Scholarship, Vienna, VA, USA).

### 2.4. Study Selection

All retrieved records were screened independently by two reviewers (A.-M.S. and A.A.) in a two-stage process. In the first stage, titles and abstracts were assessed for potential eligibility against the predefined PICO criteria. In the second stage, full texts of all potentially eligible records were retrieved and reviewed independently by the same two reviewers. Disagreements at either stage were resolved through discussion and consensus; where agreement could not be reached, a third independent reviewer (N.-C.B.) adjudicated. However, the inter-rater reliability measured by Cohen’s Kappa was 0.82, indicating a high level of agreement.

### 2.5. Data Extraction

Data extraction was performed independently by two reviewers (N.-I.V. and I.C.B.) using a piloted standardized extraction form. Extracted data included study identifiers (first author, year, country, study design, and setting), sample size and number of tumours per site, patient and tumour characteristics, specimen type, p16 IHC details (antibody clone, platform, scoring system, and positivity threshold), HPV assay details (assay type, target gene or region, HPV genotypes targeted, and positivity threshold), and 2 × 2 contingency table data. Where 2 × 2 data were not directly reported but could be calculated from reported proportions and sample sizes, this was performed and documented. Corresponding authors were contacted for missing key 2 × 2 data where studies appeared otherwise eligible.

### 2.6. Risk of Bias Assessment

Risk of bias was assessed independently by two reviewers (M.S.-F. and B.H.). For diagnostic accuracy studies, the Quality Assessment of Diagnostic Accuracy Studies 2 (QUADAS-2) tool (University of Bristol, Bristol, UK) [36] was used, evaluating four domains: patient selection, index test, reference standard, and flow and timing. Applicability concerns were assessed for the first three domains. Disagreements were resolved by consensus. Risk-of-bias judgements were visualized using the robvis tool (UK, Bristol, 4.0 International, version 22 August 2019), and the figures can be found in Supplementary Figures S1 and S2.

### 2.7. Data Synthesis

Given the observational and cross-sectional nature of the included studies and anticipated heterogeneity in p16 positivity criteria, HPV assay types, and study populations, a narrative synthesis approach was adopted throughout. No statistical pooling was performed.

## 3. Results

**Figure 1:**
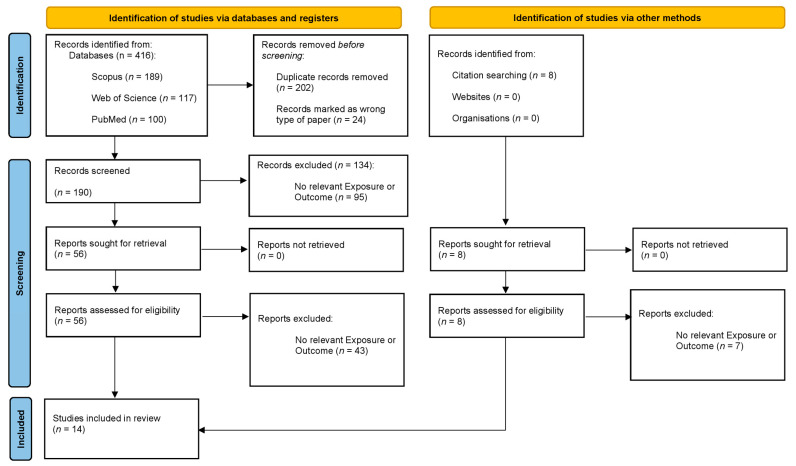
PRISMA flowchart.

The systematic search was conducted across three electronic databases—Scopus (n = 189), Web of Science (n = 117), and PubMed (n = 110)—yielding a total of 416 records. Before screening, 202 duplicate records were removed, and a further 24 records were marked as the wrong type of paper (e.g., posters, conference abstracts), leaving 190 records for title and abstract screening. Of these, 134 were excluded due to irrelevant population, no relevant exposure or outcome (n = 95), or wrong study design. The remaining 56 reports were sought for retrieval, all of which were successfully obtained and assessed for full-text eligibility. Following full-text review, 43 reports were excluded for irrelevant exposure or outcome. Additionally, citation searching identified eight further records, of which eight were assessed for eligibility, and seven were subsequently excluded for no relevant exposure or outcome. Ultimately, 14 studies were included in the review, as detailed in Figure 1.

The systematic review included 14 studies assessing p16 immunohistochemistry and HPV detection in laryngeal squamous cell carcinoma (LSCC). Sample sizes ranged from 29 LSCC cases in the basaloid SCC subgroup to 324 cases in the large cohort. Most studies were retrospective, with one prospective observational study, and were conducted across diverse regions, including Australia, the USA, China, South Africa, Japan, Jordan, Korea, Latvia, Mexico, Austria, and a multinational cohort.

Across studies, p16 cutoffs varied substantially, ranging from ≥30% moderate/strong staining to stricter thresholds such as ≥70% or ≥75% diffuse nuclear and cytoplasmic staining. HPV reference methods also differed and included HPV DNA PCR, genotyping, HPV DNA ISH, and HPV E6/E7 mRNA ISH. This methodological variation contributed to heterogeneity in reported concordance between p16 and HPV status.

Overall, the included studies mainly focused on the relationship between p16 immunohistochemical expression and molecular evidence of HPV infection in laryngeal squamous cell carcinoma. Most studies assessed whether p16 could serve as a surrogate marker for HPV-driven LSCC, while several also examined its potential prognostic value. Across the studies, HPV positivity was generally uncommon, and the agreement between p16 expression and molecular HPV testing was often weak or only fair. Prognostic findings were inconsistent, with some studies suggesting improved outcomes in HPV-positive or p16-positive subgroups, while others found no clear survival advantage. Taken together, the studies suggest that p16 expression alone is insufficient to determine HPV involvement in LSCC, and that molecular HPV testing, particularly RNA-based assays where available, is more appropriate for identifying truly HPV-driven tumours. Table 1 provides an overview of the studies included in this systematic review.

### 3.1. p16 Immunohistochemistry Scoring and Positivity Thresholds

Eight studies reported p16 immunohistochemistry methods or scoring criteria in sufficient detail for comparison. Considerable methodological variation was observed across studies, particularly in the definition of p16 positivity. Some studies applied percentage-based thresholds similar to those used in oropharyngeal squamous cell carcinoma, including ≥75% tumour expression in Kiyuna et al. [24], ≥75% stained cells in Vazquez-Guillen et al. [45], and >70% strong nuclear and cytoplasmic expression in Tannenbaum et al. [46]. In contrast, Young et al. [21] used a lower cutoff, defining positivity as moderate or strong staining in ≥30% of tumour cells, while Mena et al. evaluated several thresholds, including >25%, >50%, and ≥70%, allowing assessment of the impact of cutoff selection on concordance with HPV E6*I mRNA [20]. Other studies used alternative scoring systems: Cui et al. [23] applied a semi-quantitative immunoreactive score, with p16 positivity defined as a score ≥4, while Al-Qudah et al. [41] used a block-staining pattern based on strong nuclear and cytoplasmic expression in continuous cell segments. Overall, these findings show that p16 assessment in invasive LSCC was not standardized across studies. This heterogeneity in antibody platforms, scoring systems, and positivity thresholds may partly explain the inconsistent diagnostic performance of p16 as a surrogate marker for high-risk HPV in laryngeal squamous cell carcinoma. Table 2 summarizes the p16 immunohistochemistry protocols, scoring approaches, and positivity thresholds used across the included LSCC studies, highlighting the methodological variability in p16 assessment.

### 3.2. Diagnostic Performance of p16 Immunohistochemistry by Reference-Standard Type

Nine studies compared p16 immunohistochemistry with HPV DNA-based detection methods in LSCC, including PCR, HPV genotyping, line-probe assays, and multiplex PCR approaches. Overall, the studies showed inconsistent agreement between p16 expression and HPV DNA positivity. In several cohorts, HPV DNA positivity was more frequent than p16 positivity. For example, Kiyuna et al. reported HR-HPV DNA in 16/88 cases, but p16 overexpression in only 5/88 cases, with 11 HR-HPV DNA-positive tumours lacking p16 overexpression [24]. Similarly, Lifsics et al. found HPV16 in 22/41 LSCC cases, whereas p16 overexpression was present in only a minority of tumours, and in their later cohort p16 was detected in only 6/41 LSCC cases despite HPV DNA positivity in 32/41 [44].

Other studies demonstrated the opposite pattern, where p16 positivity exceeded HPV DNA detection. Lam et al. detected HPV DNA in only 1/85 LSCC cases, whereas p16 overexpression was observed in 11/85 cases, suggesting that p16 expression may occur independently of HPV in laryngeal tumours [39]. Sekee et al. reported HPV DNA in 4/79 laryngeal carcinomas (5.1%), comprising three high-risk types (HPV16, HPV31, HPV45) and one low-risk type (HPV11), with p16 positivity in 11/79 cases (13.9%). All three HR-HPV-positive laryngeal tumours were also p16-positive; however, the HPV11-positive case was p16-negative. Agreement between total HPV DNA positivity and p16 in the laryngeal subgroup was only fair (κ = 0.352) [40]. Hernandez et al. further showed limited overlap, with p16 expression present in only 2/16 HPV DNA-positive laryngeal cancers [37].

A few studies reported similar overall HPV DNA and p16 positivity rates but still concluded that p16 had limited diagnostic value. Al-Qudah et al. found both HPV PCR positivity and p16 positivity in 15.4% of cases yet concluded that p16 was of limited use for identifying HPV-positive LSCC [41]. Vazquez-Guillen et al. reported high rates of both HPV DNA and p16 expression, but p16 was not significantly associated with HPV status, supporting the view that p16 is not a reliable surrogate marker in LSCC [45]. Kim et al. found HR-HPV DNA in 5/43 (11.6%) laryngeal SCCs tested by PCR. p16 IHC was performed on 154 laryngeal cases, of which 11 (7.1%) were positive. In the subset of 42 laryngeal cases tested with both assays, p16 positivity was present in four cases and HR-HPV DNA positivity in five cases, with only two cases positive for both (sensitivity 40.0%, specificity 94.6%, PPV 50.0%, NPV 92.1%). These findings confirmed poor concordance between p16 and HR-HPV status in laryngeal SCC, in contrast to the high concordance demonstrated in oropharyngeal and tonsillar carcinomas in the same dataset (sensitivity 98.6% and 99.2%, respectively) [42].

Four studies assessed p16 immunohistochemistry against markers of transcriptionally active HPV infection in laryngeal carcinoma, using either HPV E6/E7 mRNA RNA in situ hybridization or E6*I mRNA detection. These studies are particularly important because RNA-based assays provide stronger evidence of biologically active HPV-driven carcinogenesis than HPV DNA detection alone. Young et al. evaluated 324 LSCC patients and found that p16 positivity was uncommon, occurring in 20/307 assessable cases [21]. HPV RNA ISH was evaluable in 80 cases, among which only seven were HPV RNA-positive; all seven were p16-positive. However, among the 14 evaluable p16-positive cases, only seven were HPV RNA-positive, showing that p16 positivity did not consistently indicate transcriptionally active HPV infection.

Similarly, Mena et al. investigated 51 HPV-DNA-positive laryngeal carcinomas from the ICO international cohort and compared p16INK4a expression with E6I mRNA as the reference standard [20]. They evaluated multiple p16 cutoffs, including >25%, >50%, and ≥70%, and found that concordance in laryngeal carcinoma was substantially weaker than in oropharyngeal carcinoma. At the ≥70% cutoff, concordance between p16 and E6I mRNA in laryngeal carcinoma was only 56.9%, supporting the conclusion that HPV-DNA testing followed by p16 immunohistochemistry may be less useful for identifying HPV-driven laryngeal carcinoma than for oropharyngeal carcinoma.

Two additional studies further highlighted the limited specificity of p16 for transcriptionally active HPV in laryngeal tumours [23,46]. Cui et al. studied 29 cases of laryngeal basaloid squamous cell carcinoma and found p16 positivity in 7/29 cases and HPV DNA positivity in 8/29 cases, but no case demonstrated transcriptionally active HPV by E6/E7 mRNA RNA ISH [23]. Tannenbaum et al. assessed 123 laryngeal SCCs using high-risk HPV E6/E7 RNA ISH and found only one HPV-associated laryngeal SCC; although this case was p16-positive, three HPV-independent laryngeal SCCs were also p16-positive, resulting in a low positive predictive value for p16 in the larynx [46].

Diagnostic accuracy could be reconstructed for nine of the fourteen studies, in which paired p16 and molecular HPV counts were reported or recoverable; the resulting 2 × 2-derived estimates, with Wilson 95% confidence intervals, are presented in Table 3, stratified by reference-standard type. For the remaining five studies, a valid 2 × 2 table could not be constructed: Lam et al. reported no high-risk HPV-positive laryngeal case [39]; Al-Qudah et al. reported only marginal totals without the p16 × HPV cross-tabulation [41]; Mena et al. reported concordance within an HPV-DNA-positive–selected population [20]; Dahm et al. pooled laryngeal and hypopharyngeal cases without a numerically defined p16 threshold [38]; and Lifsics et al. (2021) reported p16 positivity only qualitatively [43]. Across the two studies using transcriptionally active (RNA-based) reference standards, p16 sensitivity was high (Young et al., 100%, 95% CI 65–100; Tannenbaum et al., 100%, 21–100), but positive predictive value was low (50.0% and 25.0%, respectively), reflecting frequent p16 positivity in the absence of active HPV transcription. Against DNA-based reference standards, estimates were markedly heterogeneous: sensitivity ranged from 12.5% (Hernandez et al.) to 100% (Sekee et al.) and PPV from 25.0% (Hernandez et al.) to 100% (Kiyuna et al.), with no consistent pattern across cohorts. Confidence intervals were wide throughout, reflecting the small number of HPV-positive laryngeal cases in every cohort. These estimates should be interpreted cautiously: accuracy computed against HPV DNA references reflects agreement with an imperfect indicator of transforming infection rather than true diagnostic performance, so the RNA-based comparators are the more biologically meaningful, and both indicate limited positive predictive value for p16 in the larynx.

Overall, and as summarized in Table 3, p16 immunohistochemistry showed limited and inconsistent performance against both DNA-based and transcriptionally active HPV reference standards in invasive LSCC. The discordance was bidirectional: some cohorts contained HPV-positive/p16-negative tumours, while others contained p16-positive/HPV-negative tumours. This suggests that HPV DNA detection alone may capture incidental or non-transforming infection, whereas p16 overexpression may arise through HPV-independent mechanisms in laryngeal carcinogenesis. Although HPV RNA-positive tumours were almost always p16-positive (preserving sensitivity), many p16-positive tumours lacked evidence of active HPV transcription, so p16 retained only limited specificity and positive predictive value for HPV-driven LSCC. Accordingly, p16 should not be relied upon as a standalone surrogate marker for either HPV DNA positivity or transcriptionally active high-risk HPV infection in invasive laryngeal squamous cell carcinoma.

### 3.3. Patterns of p16/HPV Concordance and Discordance

Across the included studies, concordance between p16 immunohistochemistry and HPV detection in LSCC was inconsistent. Two main discordance patterns were repeatedly observed: p16-positive/HPV-negative tumours, suggesting HPV-independent p16 overexpression, and HPV-positive/p16-negative tumours, suggesting either incidental HPV detection, low viral activity, or failure of p16 to identify some HPV-positive cases. This bidirectional discordance was seen across both DNA-based and RNA-based HPV comparator studies, supporting the view that p16 cannot be interpreted as a reliable standalone surrogate marker for HPV involvement in invasive LSCC.

In studies using transcriptionally active HPV markers, discordance was particularly relevant because RNA-based assays more directly reflect biologically active HPV infection. Mena et al. found that concordance between p16 and E6I mRNA was substantially weaker in laryngeal carcinoma than in oropharyngeal and oral cavity carcinoma [20]. The authors reported that the reduction in concordance in LC was mainly driven by fewer triple-positive cases and a higher proportion of HPV-DNA-positive/p16-negative/E6I mRNA-positive tumours, which they interpreted as evidence that p16 testing, even in combination with HPV DNA, was not useful for diagnosing HPV-driven LC. Similarly, Young et al. found that all HPV RNA ISH-positive tumours were p16-positive, but not all p16-positive tumours were RNA-positive, indicating that p16 may detect some transcriptionally active HPV-positive cases while still generating false-positive results [21]. Tannenbaum et al. also showed this limitation: only one of 123 laryngeal SCCs was HPV-associated by E6/E7 RNA ISH and p16-positive, whereas three HPV-independent laryngeal tumours were also p16-positive [46].

DNA-based studies showed even more heterogeneous concordance patterns. In Lam et al., HPV DNA was detected in only one of 85 LSCC cases, whereas p16 overexpression was present in 11 cases, indicating that p16 positivity can occur in the absence of detectable HPV DNA [39]. Hernandez et al. found the opposite pattern in many tumours: p16 expression was present in only 2 of 16 HPV DNA-positive laryngeal cancer cases, while 14 HPV DNA-positive tumours lacked p16 expression [37]. The authors concluded that fewer than 10% of laryngeal tumours expressed p16 and that only approximately 2% were double-positive for p16 and HPV DNA. In Sekee et al., four of 79 laryngeal carcinomas were HPV-positive, and eleven were p16-positive, with only fair agreement between HPV DNA and p16 in laryngeal carcinoma [40]. Vazquez-Guillen et al. similarly found that p16 positivity was not significantly associated with HPV status, despite relatively high rates of both HPV detection and p16 expression [45].

Overall, these concordance and discordance patterns indicate that p16 has limited diagnostic reliability in LSCC. The p16-positive/HPV-negative pattern suggests that p16 overexpression may arise through HPV-independent alterations of the pRb/CDKN2A pathway, while the HPV-positive/p16-negative pattern suggests that HPV DNA detection may not necessarily represent transcriptionally active, biologically relevant infection. Therefore, p16 IHC should not be used alone to classify invasive laryngeal squamous cell carcinoma as HPV-driven; confirmatory HPV-specific testing, preferably using transcriptionally active HPV markers, is required when HPV attribution is clinically or analytically important. Table 4 summarizes concordant and discordant p16/HPV patterns across included LSCC studies, distinguishing p16-positive/HPV-negative and HPV-positive/p16-negative profiles.

### 3.4. Risk of Bias

Risk of bias was assessed with QUADAS-2 across all 14 included studies; the traffic-light and summary plots are provided in Supplementary Figures S1 and S2. For patient selection, one study was at low risk, eight raised some concerns, and five were at high risk of bias; concerns in this domain arose predominantly from the retrospective, single-institution designs, which raise the possibility of non-consecutive or selective case inclusion. For the index test (p16 IHC), no study was at low risk, nine raised some concerns, and five were at high risk, chiefly because a positivity threshold was pre-specified in only a subset of studies and blinding of p16 interpretation to HPV status was infrequently reported. The reference-standard domain was the most uniformly affected: all 14 studies raised some concerns, and none reached low risk, reflecting reliance on HPV DNA detection rather than a transcription-based assay in 11 studies—an imperfect indicator of biologically active HPV. For flow and timing, one study was at low risk, ten raised some concerns, and three were at high risk. Overall, 9 of the 14 studies were judged at high risk of bias and 5 at some concerns, with none at low risk. These patterns reinforce the cautious interpretation of the accuracy estimates in Table 3.

## 4. Discussion

The diagnostic accuracy of p16 immunohistochemistry as a surrogate marker for high-risk HPV infection in laryngeal squamous cell carcinoma has important implications for tumour classification, prognostic counselling, and eligibility for emerging HPV-directed treatment strategies. This systematic review synthesizes evidence from 14 studies and shows that p16 IHC performs inconsistently as a surrogate marker for HPV positivity in invasive LSCC, with bidirectional discordance observed across both DNA-based and RNA-based HPV reference standards. These findings are in contrast to the well-validated place-holder relationship established in oropharyngeal SCC and have direct relevance for how p16 should—and should not—be applied in laryngeal tumour assessment.

### 4.1. p16 as a Surrogate Marker in LSCC: A Limited and Inconsistent Relationship

Across the included studies, concordance between p16 immunohistochemistry and molecular HPV detection in invasive LSCC was limited and inconsistent, irrespective of the HPV reference standard used. Both discordance directions were observed: p16-positive/HPV-negative tumours, suggesting HPV-independent overexpression of p16, and HPV-positive/p16-negative tumours, suggesting that HPV DNA detected in laryngeal tissue may not represent transcriptionally active, biologically relevant infection [37,39,40,45]. This bidirectional pattern is biologically important because it indicates that neither false-positive nor false-negative classification is a rare event in this anatomical site—both types of misclassifications occur at clinically relevant frequencies.

The contrast with oropharyngeal SCC is instructive. In that setting, where HPV16 is the dominant oncogenic driver and the viral mechanism of p16 upregulation through E7-mediated pRb inactivation is reliably operative, pooled sensitivity and specificity of p16 IHC exceed 94% and 90%, respectively [11,47]. In laryngeal SCC, no comparable performance has been demonstrated. The findings of Mena et al. are particularly informative in this regard: directly comparing concordance across anatomical sites within the same dataset, they found concordance between p16 and E6*I mRNA in laryngeal carcinoma to be only 56.9% at the ≥70% cutoff—substantially weaker than in oropharyngeal and oral cavity carcinoma [20]. Similarly, Tannenbaum et al., using the RNA-based reference standard (HPV E6/E7 RNA ISH), identified only one HPV-associated laryngeal SCC among 123 cases, while three HPV-independent laryngeal SCCs were also p16-positive, yielding a low positive predictive value for the marker at this site [46].

These findings indicate that p16 immunohistochemistry should not be interpreted as a reliable standalone surrogate marker for HPV-driven carcinogenesis in invasive laryngeal squamous cell carcinoma. This conclusion applies across both DNA-based and RNA-based HPV reference frameworks, though the evidence is most compelling and clinically interpretable for RNA-based comparators, which more directly reflect biologically active, transforming HPV infection.

### 4.2. Biological Basis for p16/HPV Discordance in the Larynx

The discordance between p16 expression and HPV activity in laryngeal SCC is biologically expected when the distinct carcinogenic environment of the larynx is considered. Unlike the oropharyngeal crypt epithelium—which harbours specific susceptibility to HPV-driven transformation and where the viral E7 oncoprotein reliably drives compensatory p16 overexpression through pRb inactivation—the stratified squamous epithelium of the larynx is predominantly transformed by tobacco- and alcohol-related mutagenesis [5,48,49,50]. In this carcinogenic context, the CDK4/6–pRb–E2F regulatory axis can be disrupted through multiple HPV-independent mechanisms, including CDKN2A promoter demethylation, RB1 mutation or deletion, and CCND1 amplification, each of which can produce diffuse p16 overexpression in the absence of any viral oncogenic activity [5,47,48,51].

Conversely, the HPV-positive/p16-negative pattern observed in several DNA-based comparator studies is also biologically coherent [20,52,53]. HPV DNA detected in laryngeal tumour tissue may represent incidental or non-transforming infection—a well-recognized phenomenon in head and neck mucosal sites—where the virus is present but not integrated, not transcriptionally active, and not contributing to carcinogenesis [4,54].

In such cases, the E7-mediated pRb inactivation cascade is not operative, and the biological trigger for p16 upregulation is absent, despite DNA positivity. The findings of Cui et al. illustrate this mechanism precisely: among 29 laryngeal basaloid SCCs, p16 positivity and HPV DNA positivity were both observed in a minority of cases, but no case demonstrated transcriptionally active HPV by E6/E7 mRNA RNA ISH, indicating that neither DNA detection nor p16 expression reflected true HPV-driven disease [23,55]. This distinction has direct interpretive consequences for the present review: studies using HPV DNA as the reference standard cannot distinguish transforming from incidental infection, so their p16 accuracy estimates conflate two biologically different targets, whereas the RNA-based studies—although fewer—assess p16 against the clinically relevant transcriptionally active phenotype and therefore provide the more meaningful, if less precise, performance data.

This biological framework explains why RNA-based HPV reference standards are particularly important in laryngeal SCC research, and why studies relying exclusively on HPV DNA detection are likely to both overestimate the prevalence of HPV-driven disease and mischaracterize the diagnostic performance of p16 at this site.

### 4.3. Impact of p16 Positivity Threshold and Methodological Heterogeneity

A significant source of heterogeneity across the included studies was the variation in p16 positivity thresholds and scoring systems applied to laryngeal tumour specimens. Positivity criteria ranged from ≥30% moderate or strong staining [21] to ≥75% diffuse expression with intensity requirements [24,45], with intermediate cutoffs of >70% [46] and multiple evaluated thresholds of >25%, >50%, and ≥70% within a single study [20]. Additional variation was introduced by differing antibody clones—including E6H4 on Ventana platforms and EPR1473 on Abcam systems—and by alternative scoring systems such as the immunoreactive score used by Cui et al.

This methodological heterogeneity matters because threshold selection directly influences the proportion of cases classified as p16-positive and therefore the apparent sensitivity and specificity of the test relative to any HPV reference standard. The analysis by Mena et al. explicitly demonstrated this effect, showing that concordance between p16 and E6*I mRNA in laryngeal carcinoma varied across the >25%, >50%, and ≥70% cutoffs evaluated—though even at the strictest threshold, concordance remained substantially below that achieved in oropharyngeal carcinoma [20]. This observation implies that the diagnostic limitations of p16 in laryngeal SCC cannot be resolved simply by adopting a stricter staining threshold; the fundamental biological dissociation between p16 expression and HPV activity at this site persists regardless of cutoff choice.

From a methodological perspective, future studies in this area should adopt the standardized p16 protocol established for oropharyngeal SCC—clone E6H4 with a ≥70% strong diffuse nuclear and cytoplasmic staining threshold—to permit cross-study comparability, even if this threshold is ultimately shown to be insufficient for laryngeal tumour classification.

### 4.4. Clinical Implications: What p16 Can and Cannot Tell Us in Laryngeal SCC

The clinical implications of the findings are directly relevant to tumour board decision-making, molecular staging, and patient counselling in laryngeal SCC. The accumulated evidence supports a clear practical conclusion: p16 immunohistochemistry should not be used as a standalone classifier of HPV-driven disease in invasive laryngeal squamous cell carcinoma, and results should not be interpreted using the same diagnostic framework applied to oropharyngeal SCC.

This distinction matters clinically for several reasons. First, misclassifying a tobacco-driven laryngeal SCC as HPV-positive based solely on p16 overexpression could potentially expose patients to inappropriate management pathways, including de-escalation strategies currently under investigation for genuinely HPV-driven head and neck tumours [56]. Second, as molecular tumour boards increasingly incorporate HPV status into treatment planning, an erroneous HPV-positive classification derived from p16 alone may lead to prognostic miscommunication and misdirected therapeutic strategies. Third, the low prevalence of transcriptionally active HPV in laryngeal SCC—estimated at fewer than 5% of cases across studies incorporating RNA-based reference standards—means that the positive predictive value of p16 IHC in this setting is inherently limited, even when test sensitivity is adequate, reflecting the mathematical constraints of applying any marker in a low-prevalence population [25,46].

When HPV attribution in laryngeal SCC is clinically or analytically important, a sequential testing algorithm is recommended: p16 immunohistochemistry may serve as an initial screening step, but any p16-positive result should be confirmed with a tumour-based RNA-based assay—preferably HPV E6/E7 RNA ISH or E6/E7 mRNA RT-PCR—before HPV-driven disease is formally attributed. This two-step approach mirrors evolving recommendations for HPV testing in non-oropharyngeal head and neck sites and is consistent with the pre-specified reference standard hierarchy applied in this review.

### 4.5. Comparison with Existing Evidence

To our knowledge, prior systematic reviews have addressed p16 performance in oropharyngeal SCC and HPV prevalence across head and neck sites [11], but none have focused on laryngeal-specific diagnostic accuracy with paired p16 and molecular HPV data as the organizing framework.

The finding that HPV-driven laryngeal SCC represents transcriptionally active HPV appears to account for a small minority of cases—consistently below 5% in studies applying RNA-based reference standards—underscoring the biological and clinical distinctions between laryngeal and oropharyngeal SCC.

### 4.6. Strengths and Limitations

This systematic review has several methodological strengths. The use of a PRISMA-compliant framework with prospective PROSPERO registration, a pre-specified reference standard hierarchy prioritizing RNA-based assays, and a structured two-reviewer process for study selection, data extraction, and risk of bias assessment contributes to the transparency and reproducibility of the review. The inclusion of studies using both DNA-based and RNA-based HPV reference standards allowed meaningful subgroup comparison of p16 performance across biologically distinct HPV testing frameworks, which is a key analytical advantage over prior narrative syntheses.

Several limitations must be acknowledged. The decision to adopt a synthesis approach rather than statistical pooling was driven by the substantial heterogeneity in p16 positivity thresholds, HPV assay types, and study populations—conditions under which meta-analytic pooling of sensitivity and specificity estimates would produce misleading summary statistics. While this decision is methodologically justified, it limits the precision of the review’s conclusions. For the same reason, a formal GRADE assessment of certainty for diagnostic tests was not undertaken. GRADE for diagnostic accuracy is designed to rate confidence in a target accuracy estimate—typically a pooled sensitivity–specificity pair—whereas the heterogeneous and non-equivalent reference standards (DNA-based versus transcriptionally active) and the small number of HPV-positive laryngeal cases per study precluded valid pooling. Applying GRADE across such non-comparable estimands would have yielded a uniform very-low-certainty rating that adds little beyond the QUADAS-2 findings already reported; the risk-of-bias and heterogeneity considerations summarized here should therefore be read as the qualitative equivalent of a low-certainty judgement. The number of studies using RNA-based reference standards—the most biologically informative comparator for p16 in this setting—remains small (four studies), restricting the strength of inferences in this critical subgroup. The predominance of retrospective study designs across the included literature, as confirmed by QUADAS-2 assessment, introduces risks of patient selection bias, incomplete covariate adjustment, and referral bias that cannot be fully mitigated through methodological controls. Heterogeneity in p16 antibody clones, automated platforms, and scoring systems precluded direct cross-study comparability of p16 assay performance and may have contributed to variability in reported concordance estimates. Formal assessment of publication bias was not feasible given the small number of studies per subgroup; the possibility that studies reporting poor p16 performance were more likely to be published in the context of a site where HPV prevalence is low cannot be excluded.

A notable limitation concerns the partial overlap between two studies included. Lifsics et al. (2021) [43] and Lifsics et al. (2023) [44] share an identical cohort of 41 LSCC patients from the same institution and recruitment period, with the 2023 publication expanding the original dataset through the addition of an oropharyngeal subgroup, survival analysis, p53 immunohistochemistry, and HPV16 E6/E7 mRNA detection. To mitigate the risk of data duplication, the two papers were treated as a paired dataset rather than independent studies, each contributing non-redundant data to distinct aspects of the synthesis. Nevertheless, this overlap represents an inherent methodological constraint that should be considered when interpreting the findings of this review.

The exclusion of non-English-language publications represents a potential source of selection bias. Given that several included studies originate from non-Anglophone research environments—including China, Japan, Latvia, Jordan, Mexico, and South Korea—it is possible that relevant studies published in those languages were not captured by the search strategy, potentially skewing the geographic and epidemiological representation of the evidence base.

### 4.7. Future Research Directions

Several research priorities emerge from this review. First, prospective studies incorporating standardized p16 protocols—specifically clone E6H4 at a ≥70% diffuse staining threshold—alongside HPV E6/E7 RNA ISH as the reference standard are required to generate laryngeal-site-specific diagnostic accuracy estimates with sufficient precision for clinical guideline development. Second, multicentre registry-based studies with adequate sample size are needed to characterize the prevalence and clinical phenotype of truly HPV-driven laryngeal SCC and to determine whether this molecular subtype carries the prognostic advantage observed in oropharyngeal SCC. Third, investigation of alternative or complementary biomarkers for HPV attribution in laryngeal SCC—including HPV serology, viral load quantification, and methylation-based assays—may identify more reliable surrogates than p16 alone for this anatomical site. Finally, it would be of interest to evaluate whether HPV-driven laryngeal SCC, when accurately identified using RNA-based standards, shares the clinical characteristics and treatment responsiveness reported for HPV-driven oropharyngeal SCC, which remains a critical unanswered question for the field.

## 5. Conclusions

This systematic review of 14 studies shows that p16 immunohistochemistry should not be relied upon as a standalone surrogate marker for high-risk HPV-driven carcinogenesis in invasive laryngeal squamous cell carcinoma. Across studies, p16/HPV discordance was frequent and bidirectional, including both p16-positive/HPV-negative and HPV-positive/p16-negative tumours, even when RNA-based reference standards were used. These findings contrast with the established role of p16 in oropharyngeal SCC and reflect the distinct biology of laryngeal SCC, where tobacco- and alcohol-related carcinogenesis predominates, and transcriptionally active HPV appears to account for only a small minority of cases. Although methodological heterogeneity across studies limits definitive pooled accuracy estimates, the available evidence supports a clinically relevant conclusion: p16 IHC should not be used alone to classify invasive laryngeal SCC as HPV-driven. When HPV attribution is clinically or analytically important, p16-positive cases should be confirmed using tumour-based HPV-specific testing, preferably RNA-based assays such as HPV E6/E7 RNA ISH or E6/E7 mRNA RT-PCR. Future prospective studies using standardized p16 protocols and RNA-based HPV reference standards are needed to generate larynx-specific diagnostic accuracy estimates and inform clinical guidelines.

## Figures and Tables

**Table 1:** Overview of the selected studies.

First Author, Year	Country	Design	n (LSCC)	p16 Cutoff	HPV Reference Method	Main Finding on p16	Reference
Young 2015	Australia	Retrospective cohort	324	≥30% moderate/strong staining	HPV E6/E7 mRNA ISH	p16 overexpression infrequent; poor prognostic utility in LSCC	[21]
Hernandez 2016	USA	Population-based retrospective study	101	p16 expression by IHC	HPV DNA genotyping	p16 not reliable surrogate for HPV in laryngeal cancer	[37]
Cui 2018	China	Retrospective study	29 (BSCC subtype)	p16 IHC	HPV DNA PCR + E6/E7 mRNA ISH	HPV not correlated with p16 expression	[23]
Dahm 2018	Austria	Retrospective study	85	p16 IHC (CINtec)	HPV ISH	No survival advantage for p16-positive LSCC	[38]
Lam 2018	China	Retrospective cross-sectional	85	p16 overexpression by IHC	HPV DNA PCR	p16 not a surrogate marker for HPV in LSCC	[39]
Sekee 2018	South Africa	Prospective observational study	79	p16 immunohistochemistry	HPV PCR assays targeting L1/E6	Only fair agreement between p16 and HPV status in LSCC	[40]
Kiyuna 2019	Japan	Retrospective study	88	≥75% diffuse staining with moderate intensity	HR-HPV DNA PCR	HPV+/p16+ cases showed better survival trend	[24]
Al-Quadah 2020	Jordan	Retrospective study	52	p16 immunostaining	HPV PCR	Limited utility of p16 for identifying HPV in LSCC	[41]
Kim 2020	Korea	Retrospective cohort	43	p16 IHC	HR-HPV DNA PCR	Low HPV prevalence in laryngeal SCC; p16 concordance mainly validated for OPSCC	[42]
Lifsics 2021	Latvia	Retrospective study	41	p16 IHC	HPV16 DNA PCR + E6/E7 IHC	p16 impractical as surrogate marker in LSCC	[43]
Mena 2022	Multinational (29 countries)	Retrospective multicentre study	51	>25%, >50%, ≥70% nuclear/cytoplasmic staining evaluated	HPV DNA + E6*I mRNA	Fair concordance between p16 and HPV mRNA in LC; p16 not reliable alone	[20]
Lifsics 2023	Latvia	Retrospective cohort	41	p16 IHC overexpression	HPV DNA PCR	p16 associated with survival in some HNSCC subgroups but role in LSCC remained unclear	[44]
Vazquez-Guillen 2023	Mexico	Retrospective study	103	≥75% stained cells	HPV genotyping (INNO-LiPA) + EBV qPCR	p16 not reliable surrogate marker for HPV in LSCC	[45]
Tannenbaum 2024	USA	Retrospective tissue microarray study	123	>70% strong nuclear/cytoplasmic staining	HPV E6/7 RNA ISH	Very low prevalence of HPV-associated LSCC; limited PPV of p16 in larynx	[46]

LSCC: Laryngeal Squamous Cell Carcinoma; BSCC: Basaloid Squamous Cell Carcinoma; p16 IHC: p16 Immunohistochemistry; HPV: Human Papillomavirus; HR-HPV: High-Risk Human Papillomavirus; HPV DNA: Human Papillomavirus Deoxyribonucleic Acid; PCR: Polymerase Chain Reaction; ISH: In Situ Hybridization; RNA ISH: Ribonucleic Acid In Situ Hybridization; mRNA: Messenger Ribonucleic Acid; E6/E7: Human Papillomavirus Viral Oncoproteins E6 and E7; E6I mRNA: Spliced HPV E6I Messenger Ribonucleic Acid; INNO-LiPA: INNO-LiPA Human Papillomavirus Genotyping Assay; EBV: Epstein–Barr Virus; qPCR: Quantitative Polymerase Chain Reaction; LC: Laryngeal Carcinoma; HNSCC: Head and Neck Squamous Cell Carcinoma; OPSCC: Oropharyngeal Squamous Cell Carcinoma; PPV: Positive Predictive Value.

**Table 2:** p16 immunohistochemistry antibodies, protocols, scoring approaches, and positivity thresholds across the included LSCC studies.

Study	Sample/Material	p16 Antibody (Clone; Host; Clonality; Vendor)	Detection Platform/Format	Scoring Approach	Positivity Threshold	Ref.
Young 2015	324 LSCC; 307 assessable	Clone E6H4; mouse monoclonal; Ventana	Benchmark Ultra; UltraView DAB; prediluted	Nuclear + cytoplasmic; intensity 0–3, percentage 0–100%	Moderate/strong staining in ≥30% of cells	[21]
Cui 2018	29 laryngeal basaloid SCC	Cat. ZM-0205 (clone not named); mouse monoclonal; OriGene	Biotin–streptavidin DAB; prediluted	Immunoreactive score (proportion × intensity)	IRS ≥ 4	[23]
Sekee 2018	79 laryngeal SCC (fresh-frozen)	Clone E6H4 (CINtec); mouse monoclonal; Ventana	Benchmark XT; ready-to-use kit	Nuclear + cytoplasmic	≥70% moderate–strong nuclear + cytoplasmic	[40]
Dahm 2018	85 laryngeal ca.; mixed with hypopharyngeal	Clone E6H4 (CINtec); mouse monoclonal; Ventana	Benchmark Ultra; UltraView DAB; ready-to-use	Per prior HPV detection guidelines	Not numerically specified	[38]
Kiyuna 2019	88 laryngeal cancers	Clone E6H4 (CINtec); mouse monoclonal; MTM Labs/Roche	Ready-to-use kit; DAB	Diffuse staining + intensity requirement	≥75% with ≥moderate intensity	[24]
Mena 2022	51 HPV-DNA-positive laryngeal ca.	Not restated (standardized ICO protocol)	NR	Nuclear + cytoplasmic at multiple cutoffs	>25%, >50%, ≥70% evaluated	[20]
Vazquez-Guillen 2023	103 LSCC	Clone EPR1473 (ab108349); rabbit monoclonal; Abcam	HRP/DAB (ABC) kit; dilution 1:100	Percentage of stained tumour cells	≥75% stained cells	[45]
Tannenbaum 2024	123 laryngeal SCC (multi-site TMA)	Clone E6H4; mouse monoclonal; Ventana	Discovery XT; prediluted	Strong nuclear + cytoplasmic	>70% tumour cells	[46]

Antibody characteristics are reported as stated in each primary study. The immunogen and target epitope of the p16 antibody were not reported in any included study. Most studies used the mouse monoclonal clone E6H4 (the antibody in the CINtec p16 assay; Ventana/Roche), whereas Vazquez-Guillen et al. used the rabbit monoclonal clone EPR1473 (Abcam) and Cui et al. an OriGene mouse monoclonal (clone not named); Mena et al. did not restate the clone. This reagent-level heterogeneity (clone, host species, and detection format) compounds the variability in positivity thresholds and limits the direct comparability of p16 staining across studies. LSCC, laryngeal squamous cell carcinoma; TMA, tissue microarray; DAB, 3,3′-diaminobenzidine; HRP, horseradish peroxidase; IRS, immunoreactive score; ICO, Institut Català d’Oncologia cohort; NR, not reported.

**Table 3:** Diagnostic performance of p16 immunohistochemistry in invasive laryngeal squamous cell carcinoma, by reference-standard type, with study-level accuracy reconstructed from reported paired counts.

Study	Reference Standard (Type Assay)	n (Larynx)	HPV Positivity	p16 Positivity	TP/FP/FN/TN	Sens.	Spec.	PPV	NPV	Ref.
Young 2015	RNA HR-HPV E6/E7 RNA ISH ^f^	80 ^†^	7 (RNA ISH+)	20/307, 6.5%	7/7/0/66	100 (65–100)	90.4 (82–95)	50.0 (27–73)	100 (94–100)	[21]
Hernandez 2016	DNA · HPV genotyping	101	16/101, 15.8%	8/101, 7.9%	2/6/14/79	12.5 (3–36)	92.9 (85–97)	25.0 (7–59)	84.9 (76–91)	[37]
Cui 2018	DNA · PCR-RDB (+mRNA ISH) ^g^	29	8/29, 27.6% (DNA)	7/29, 24.1%	3/4/5/17	37.5 (14–69)	81.0 (60–92)	42.9 (16–75)	77.3 (57–90)	[23]
Sekee 2018	DNA · PCR (L1/E6) ^h^	79	4/79, 5.1% (3 HR)	11/79, 13.9%	3/8/0/68	100 (44–100)	89.5 (81–95)	27.3 (10–57)	100 (95–100)	[40]
Lam 2018	DNA · PCR ^a^	85	1/85, 1.2% (LR HPV-6)	11/85, 12.9%	—	Not calculable				[39]
Dahm 2018	DNA · HPV DNA (mixed cohort) ^b^	NR	NR	NR	—	Not calculable				[38]
Kiyuna 2019	DNA · HR-HPV PCR	88	16/88, 18.2%	5/88, 5.7%	5/0/11/72	31.2 (14–56)	100 (95–100)	100 (57–100)	86.7 (78–92)	[24]
Al-Qudah 2020	DNA · PCR ^c^	52	8/52, 15.4%	8/52, 15.4%	—	Not calculable				[41]
Kim 2020	DNA · HR-HPV PCR/chip	42 ^‡^	5/43, 11.6%	11/154, 7.1%	2/2/3/35	40.0 (12–77)	94.6 (82–99)	50.0 (15–85)	92.1 (79–97)	[42]
Lifsics 2021	DNA · PCR (Anyplex II) ^d^	41	HPV16 22/41, 53.7%	Low	—	Not calculable				[43]
Mena 2022	RNA · E6*I mRNA ^e^	51 ^§^	per mRNA	varies by cutoff	—	Not calculable				[20]
Vazquez-Guillen 2023	DNA · INNO-LiPA genotyping ^i^	103	57/103, 55.3% (HR 42)	55/103, 53.4%	32/23/25/23	56.1 (43–68)	50.0 (36–64)	58.2 (45–70)	47.9 (34–62)	[45]
Lifsics 2023	DNA · HPV16 PCR ^j^	41	32/41, 78.1% (HPV16 22)	6/41, 14.6%	5/1/17/18	22.7 (10–43)	94.7 (75–99)	83.3 (44–97)	51.4 (36–67)	[44]
Tannenbaum 2024	RNA · HR-HPV E6/E7 RNA ISH ^k^	123	1/123 (active)	4/123, 3.3%	1/3/0/119	100 (21–100)	97.5 (93–99)	25.0 (5–70)	100 (97–100)	[46]

Values are point estimates with Wilson 95% confidence intervals (in parentheses). HPV positivity is reported for each study’s reference method. DNA, HPV DNA-based reference; RNA, transcriptionally active (E6/E7 mRNA or RNA ISH) reference; TP/FP/FN/TN, true/false positive/negative; Sens., sensitivity; Spec., specificity; PPV/NPV, positive/negative predictive value; NR, not reported; HR, high-risk; LR, low-risk; ISH, in situ hybridization; PCR, polymerase chain reaction. Accuracy against DNA-based reference standards reflects agreement with an imperfect indicator of transforming infection and should be interpreted accordingly. ^†^ RNA-ISH-evaluable subset (80 of 307 p16-assessable). ^‡^ Subset tested with both assays. ^§^ HPV-DNA-positive-selected cohort. ^a^ No high-risk HPV-positive case (single low-risk HPV-6); Sens./PPV undefined. ^b^ Laryngeal and hypopharyngeal cases pooled; p16 threshold not numerically defined. ^c^ Marginal totals only, no p16 × HPV cross-tabulation. ^d^ p16 positivity reported qualitatively. ^e^ Concordance within an HPV-DNA-positive-selected population (≥70% cutoff). ^f^ Within the RNA-ISH-evaluable subset. ^g^ Basaloid subtype; no E6/E7 mRNA-positive case (p16 PPV for active HPV = 0%). ^h^ Reference restricted to high-risk HPV; one low-risk HPV-11 (p16-negative) case excluded. ^i^ Reference = total HPV DNA (any genotype). ^j^ Reference = HPV16 DNA; based on 5/22 HPV16-positive cases expressing p16. ^k^ Single HPV-positive case; estimate imprecise.

**Table 4:** Patterns of p16/HPV concordance and discordance in LSCC.

Study	Reference Standard	Predominant Discordance Direction	Interpretation	Ref.
Young 2015	HPV E6/E7 RNA ISH	p16+/RNA−	p16 captured all RNA-positive cases but lacked specificity	[21]
Hernandez 2016	HPV DNA genotyping	HPV DNA+/p16−	Weak p16–HPV DNA correlation; only ~2% double-positive	[37]
Cui 2018	HPV DNA + E6/E7 mRNA ISH	Bidirectional; no active HPV	Neither DNA positivity nor p16 reflected transforming HPV	[23]
Lam 2018	HPV DNA PCR	p16+/HPV−	p16 overexpression despite near-absent HPV DNA detection	[39]
Sekee 2018	HPV DNA PCR	p16+/HPV−	All HR-HPV-positive cases were p16+; overall agreement only fair (κ = 0.352)	[40]
Mena 2022	E6*I mRNA (HPV-DNA+ cases)	HPV-DNA+/p16−/mRNA+	Concordance weaker in larynx than in oropharynx/oral cavity	[20]
Vazquez-Guillen 2023	HPV DNA genotyping	Bidirectional	p16 not significantly associated with HPV status	[45]
Tannenbaum 2024	HPV E6/E7 RNA ISH	p16+/RNA−	One concordant case; three HPV-independent p16+ cases → low PPV	[46]

## Data Availability

No new data were generated for this paper.

## Supplementary Materials

The following supporting information can be downloaded at: https://www.mdpi.com/article/10.3390/medicina62071372/s1, File S1: ROB assessments plots are available. Figure S1. Risk of bias assessment: Traffic-light plot; Figure S2. Risk of bias assessment: Summary plot. File S2: PRISMA·2020-Checklist.

## Abbreviations

The following abbreviations are used in this manuscript:

AJCC	American Joint Committee on Cancer
BSCC	Basaloid Squamous Cell Carcinoma
CCND1	Cyclin D1
CDK4/6	Cyclin-Dependent Kinase 4 and 6
CDKN2A	Cyclin-Dependent Kinase Inhibitor 2A
DNA	Deoxyribonucleic Acid
DNA ISH	DNA In Situ Hybridization
E2F	E2F Transcription Factors
E6/E7	Human Papillomavirus Viral Oncoproteins E6 and E7
E6*I mRNA	Spliced HPV E6I Messenger Ribonucleic Acid
EBV	Epstein–Barr Virus
HNSCC	Head and Neck Squamous Cell Carcinoma
HPV	Human Papillomavirus
HPSCC	Hypopharyngeal Squamous Cell Carcinoma
HR-HPV	High-Risk Human Papillomavirus
ICO	Institut Català d’Oncologia
IHC	Immunohistochemistry
INNO-LiPA	INNO-LiPA Human Papillomavirus Genotyping Assay
κ	Cohen’s Kappa Coefficient
LC	Laryngeal Carcinoma
LR-HPV	Low-Risk Human Papillomavirus
LSCC	Laryngeal Squamous Cell Carcinoma
MeSH	Medical Subject Headings
mRNA	Messenger Ribonucleic Acid
NPV	Negative Predictive Value
OCC	Oral Cavity Carcinoma
OPC	Oropharyngeal Carcinoma
OPSCC	Oropharyngeal Squamous Cell Carcinoma
p16	p16INK4a/Cyclin-Dependent Kinase Inhibitor 2A protein
PCR	Polymerase Chain Reaction
PICOS	Population, Intervention, Comparator, Outcome, Study Design
PPV	Positive Predictive Value
pRb	Retinoblastoma Protein
PRISMA	Preferred Reporting Items for Systematic Reviews and Meta-Analyses
PROSPERO	International Prospective Register of Systematic Reviews
qPCR	Quantitative Polymerase Chain Reaction
QUADAS-2	Quality Assessment of Diagnostic Accuracy Studies 2
RB1	Retinoblastoma 1 Gene
RNA	Ribonucleic Acid
RNA ISH	RNA In Situ Hybridization
RT-PCR	Reverse-Transcription Polymerase Chain Reaction
SCC	Squamous Cell Carcinoma
TMA	Tissue Microarray
