# Supplementary material for: Diagnostic Accuracy of p16 Immunohistochemistry as a Marker of High-Risk HPV in Invasive Laryngeal Squamous Cell Carcinoma: A Systematic Review
Source: Medicina (Kaunas). 2026 Jul 16;62(7):1372. doi: 10.3390/medicina62071372 (PMC13414022; doi:10.3390/medicina62071372)

medicina

MDPI

Supplementary Materials

Diagnostic Accuracy of p16 Immunohistochemistry as a Marker of High-Risk HPV in Invasive Laryngeal Squamous Cell Carcinoma: A Systematic Review

Supplemental Figure S1. Risk of bias assessment: Traffic-light plot

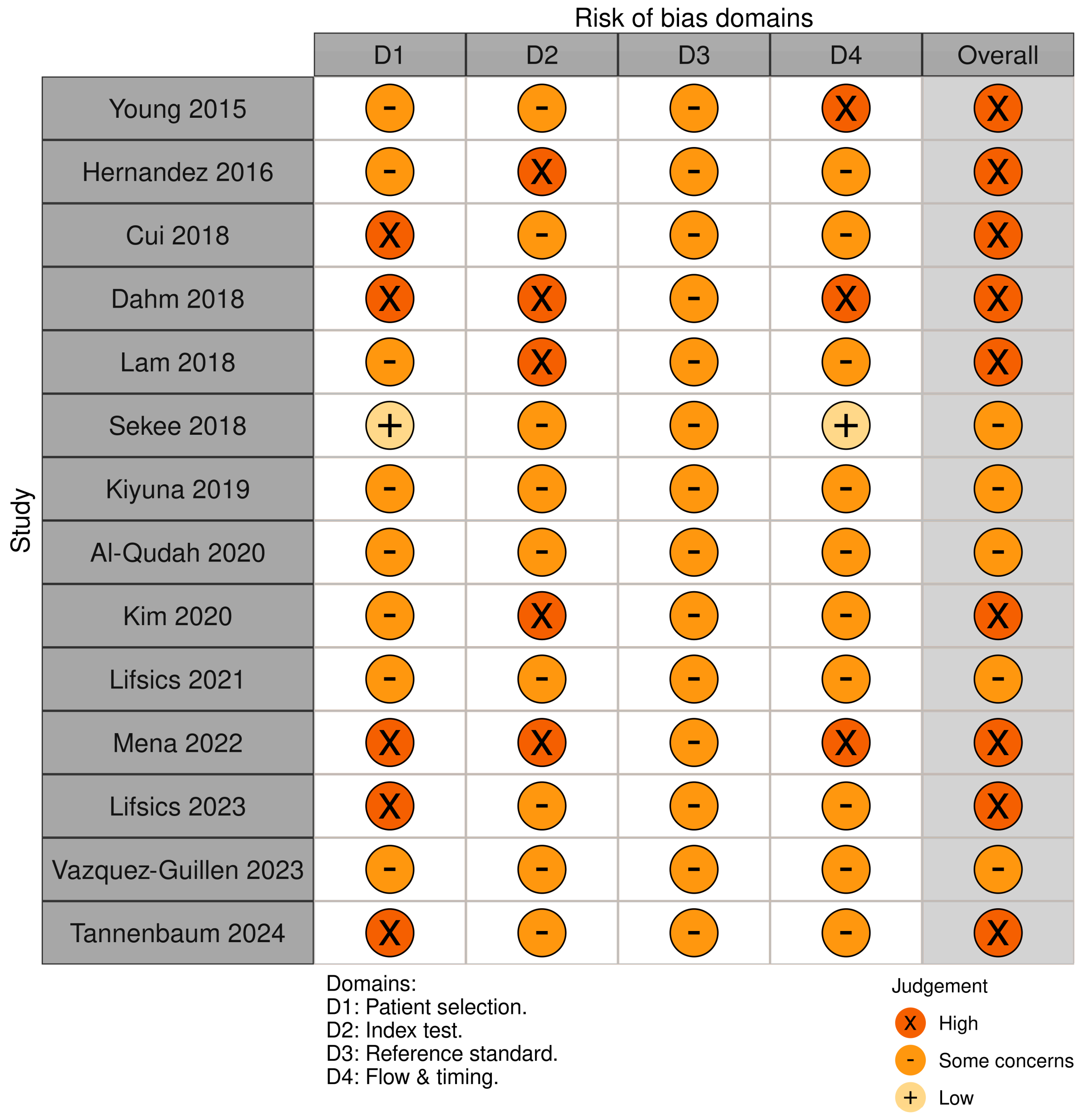

Supplemental Figure S2. Risk of bias assessment: Summary plot

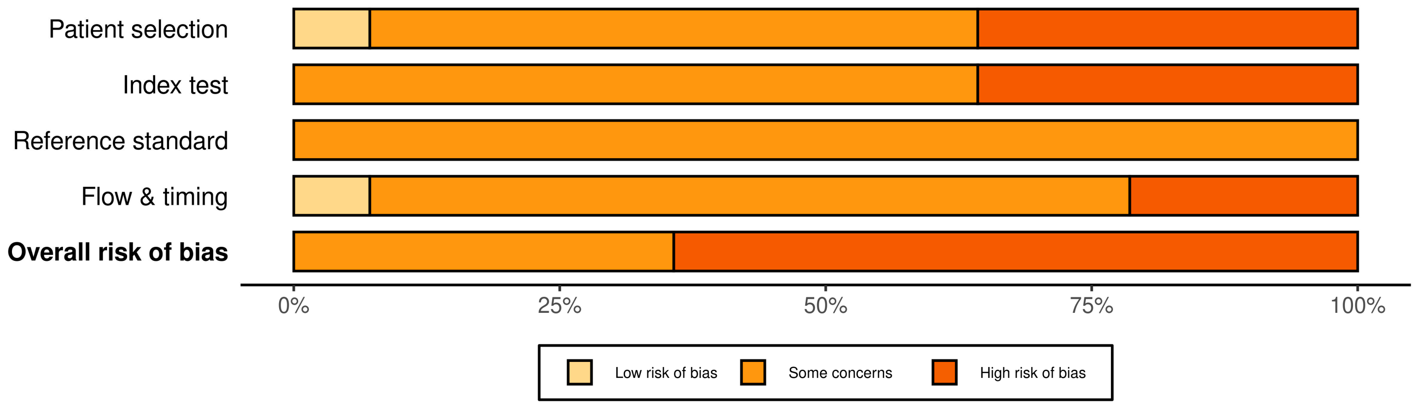

Supplement: Supplementary file 1 [file medicina-62-01372-s001.zip › Supplementary File S1. ROB.pdf]
